# The efficacy of bimatoprost ophthalmic solution combined with NB-UVB phototherapy in non-segmental and segmental vitiligo: a single-blind randomized controlled study

**DOI:** 10.1038/s41598-023-32591-8

**Published:** 2023-04-20

**Authors:** Narumol Silpa-archa, Surachanee Likittanasombat, Chalermkwan Apinuntham, Chutipon Pruksaeakanan, Norramon Charoenpipatsin, Chayada Chaiyabutr, Chanisada Wongpraparut

**Affiliations:** Department of Dermatology, Faculty of Medicine Siriraj Hospital, Mahidol University, 2 Wanglang Road, Bangkoknoi, Bangkok, 10700 Thailand

**Keywords:** Skin diseases, Pharmacology

## Abstract

Bimatoprost ophthalmic solution 0.03% (PGF2α analogues) combined with narrowband ultraviolet B (NB-UVB) was reported to be an effective treatment for vitiligo. To investigate the efficacy and safety of treatment for non-segmental/segmental vitiligo compared among bimatoprost ophthalmic solution 0.01% combined with NB-UVB phototherapy, bimatoprost monotherapy, and placebo. This single-blind randomized controlled study enrolled stable Thai vitiligo patients with at least three similarly sized lesions in the same anatomical area. The treatment duration was 6 months with 1- and 2-month post-treatment follow-ups. The 3 selected lesions on each patient were randomized to receive combination therapy, monotherapy, or placebo. The Vitiligo Area Scoring Index (VASI) was used to evaluate lesion response. Of the 25 initially enrolled subjects, 19 patients were analyzed. There were 13 and 6 non-segmental and segmental vitiligo cases, respectively. Eight and 11 cases had face/neck and non-face/neck lesions, respectively. Non-segmental vitiligo and non-face/neck vitiligo patients in the combination group had significant improvement in VASI score at 3 months, 6 months, and at the 2-month follow-up. No side effects were observed/reported. Bimatoprost combination therapy was shown to be safe and effective for treating Thai patients with non-segmental vitiligo in non-face/neck areas of the body.

## Introduction

### Background and objectives

Vitiligo is a common depigmentation disorder that is mainly caused by CD8+ T cell destruction of melanocytes. Vitiligo lesions are characterized by well-defined depigmented patches that can present at any part of the body. The prevalence of vitiligo is 0.1–2% of the worldwide population^1–4^. Three types of vitiligo include non-segmental, segmental, and unclassified vitiligo. Non-segmental vitiligo is the most common type, and it has an unpredictable clinical course. In contrast, segmental type vitiligo has a comparatively stable clinical course. Treatment of vitiligo is challenging. The goals of vitiligo treatment include stabilization and containment of active disease and stimulation of repigmentation. Current treatments include topical therapy (e.g., corticosteroids, calcineurin inhibitors), systemic therapy (e.g., corticosteroids, immunosuppressants, and antioxidants), phototherapy [e.g. narrowband ultraviolet B (NB-UVB) phototherapy, excimer laser/light, and psoralen plus UVA], and surgical methods (e.g., melanocyte-keratinocyte transplantation and lasers). NB-UVB, which is the mainstay treatment, is commonly combined with other treatments (topical, systemic, and/or surgical)^5^.

Bimatoprost ophthalmic solution 0.03% (Lumigan^®^; Allergan, Inc., Dublin, Ireland), which is a PGF2α analogue, was approved by the United States Food and Drug Administration as a treatment for open-angle glaucoma and ocular hypertension in 2001^6^. Since one of the properties of bimatoprost is the induction of melanogenesis, bimatoprost was investigated for possible benefit in vitiligo. Pharmacological and clinical studies revealed bimatoprost to be a safe and effective alternative treatment for vitiligo. However, data specifying the safety and efficacy of bimatoprost in the treatment of Thai patients with vitiligo are scarce. Moreover, bimatoprost 0.01% is the only concentration available in our institution. Accordingly, this study aimed to investigate the efficacy and safety of treatment for non-segmental or segmental vitiligo compared among bimatoprost ophthalmic solution 0.01% combined with NB-UVB phototherapy, bimatoprost monotherapy, and saline solution placebo control.

## Methods

### Trial design

This study was designed as a single-blind randomized controlled study, with three parallel group in each patient and allocation ratio of 1:1. Three vitiliginous lesions located at the same part of the body in each participant were simply randomized to receive three different treatment interventions, including bimatoprost 0.01% monotherapy, combination therapy consisting of bimatoprost 0.01%, and NB-UVB phototherapy, and normal saline solution placebo. The treatment duration was 6 months with 1- and 2-month post-treatment follow-ups. The protocol for this study was approved by the Siriraj Institutional Review Board (COA no. Si263/2016). Written informed consent was obtained from all subjects before their enrollment in the study. All methods were performed by the relevant guidelines and regulations.

### Participants

This study was conducted at the Department of Dermatology, Faculty of Medicine Siriraj Hospital, Mahidol University, Bangkok, Thailand. Thai patients aged 10 years or older with stable vitiligo and had at least three similarly sized lesions in the same anatomical area were prospectively enrolled in to the study. Patient recruitment process was performed at dermatology outpatient clinic, vitiligo clinic and phototherapy clinic. All patients had stable vitiligo which defined as no new lesion and no clinical signs of active vitiligo (confetti repigmentation, trichrome lesion, Köebner phenomenon, or inflammatory lesion) within 3 months. The recruited participants had to wash out topical corticosteroids and/or calcineurin inhibitors for 2 weeks, and had to discontinue phototherapy for 4 weeks before starting the study. Patients who were pregnant or lactating, or who had a history of skin cancer, allergy to PGF2α analogues, photosensitivity, or photodermatoses were excluded. Participants who could not follow the study protocol or who received phototherapy more than twice a week were withdrawn from the study.

### Intervention

Three vitiliginous lesions in each patient were randomized to lesions A, B or C to receive bimatoprost 0.01% monotherapy, combination therapy consisting of bimatoprost 0.01%, and NB-UVB phototherapy, and normal saline solution placebo. The bottles containing the study solutions (placebo and bimatoprost 0.01%) were all the same color and shape, and each was labeled A, B, or C to correspond with lesions A, B, and C. The lesion randomization pattern was illustrated and recorded in a case record form, and detailed written instructions were provided for each participant. All 3 study solutions (A, B, C) were applied twice daily to each respective lesion. NB-UVB was administered using a DuaLight targeted phototherapy system (Theralight LLC, Lindon, UT, USA) to the vitiligo lesion randomized to the combination therapy group. Phototherapy was performed twice weekly. The intensity of phototherapy was started at 200 mJ/cm^2^, which was followed by a 10–15% increase in intensity until the clinical endpoint (erythema within 24 h) was achieved. Digital photographs were serially taken at every one-month visit, including 6 monthly treatment period visits and 2 monthly post-treatment follow-up visits, using a Nikon D700 digital camera.

### Outcomes

The primary outcome was clinical repigmentation which was evaluated monthly by two blinded dermatologists using the Vitiligo Area Scoring Index (VASI). In summary, the VASI score is calculated by adding the percentage of vitiligo involvement at six body regions and multiplying it by the residual depigmentation. The depigmentation degree was validated as 100%, 90%, 75%, 50%, 25%, and 10%^7^. The percentage of vitiligo involvement is calculated using the palmar method. If the opinions of the two evaluating dermatologists in VASI assessment were different, a conclusion was arrived at via discussion-based consensus. The secondary outcomes included a side effect of treatment which was closely monitored at each monthly visit. The quality of repigmentation was also evaluated and defined as good color matching or hyperpigmentation.

### Sample size

The sample size for this study was calculated using data from a study conducted by Anbar, et al. in which vitiligo treatment was compared between latanoprost and placebo and latanoprost with NB-UVB and NB-UVB^8^. Using one-way repeated measure contrast for sample calculation, when the test of a single contrast at the 0.05 level in a one-way repeated measures analysis of variance with three levels will have 80% power to detect a contrast 50 (assumed VASI score of a placebo, monotherapy, and combination therapy as 90, 80 and 50), with a square root of the sum of the squared coefficients of 2.44949, assuming a standard deviation at each level of 30 and a between the level correlation of 0.1, the calculated effect size was 0.717 as shown in the formulation $\Delta =\mathrm{ICI}/[\mathrm{D\sigma SQRT}(1-\mathrm{p})]$ using nQuery Advisor version 6.0 and therefore, the calculated sample size was 17. We assumed a total dropout of 30% during the course of the study. Therefore, our sample size calculation defined 25 subjects as the minimum number needed to evaluate among placebo, monotherapy, and combination therapy in this study.

### Randomization

#### Sequence generation

Three vitiliginous lesions on the same anatomical areas in each participant were chosen and assigned for each treatment by simple randomization. A random number table of three treatment intervention (A, B, and C) was generated by computer for 25 rows.

#### Allocation concealment

Allocation concealment was performed by non-evaluating investigators using sequentially number technique.

#### Implementation

Non-evaluating investigators generated random allocation sequence, enrolled all participants and assigned participants to the interventions.

#### Blinding

Two evaluating dermatologists were blinded to the randomization process. In addition, all participants were assessed by the two blinded dermatologists before beginning the intervention and after the intervention process.

### Statistical methods

Descriptive statistics were used to summarize demographic and clinical data. Categorical data are shown as numbers and percentages, and continuous data are given as median (minimum, maximum). Repeated measures analysis of variance (ANOVA) with multiple comparison by the Bonferroni method was used to compare the primary outcome between and among the three different interventions at different time points. All statistical analyses were performed using a SPSS Statistics version 18.0 (SPSS, Inc., Chicago, IL, USA), and a *p*-value less than 0.05 was considered statistically significant for all tests.

### Registration

This study was registered in the Thai Clinical Trials Registry (registration no. TCTR20170526002 [26/05/2017]).

## Results

### Participant flow

**Figure 1:**
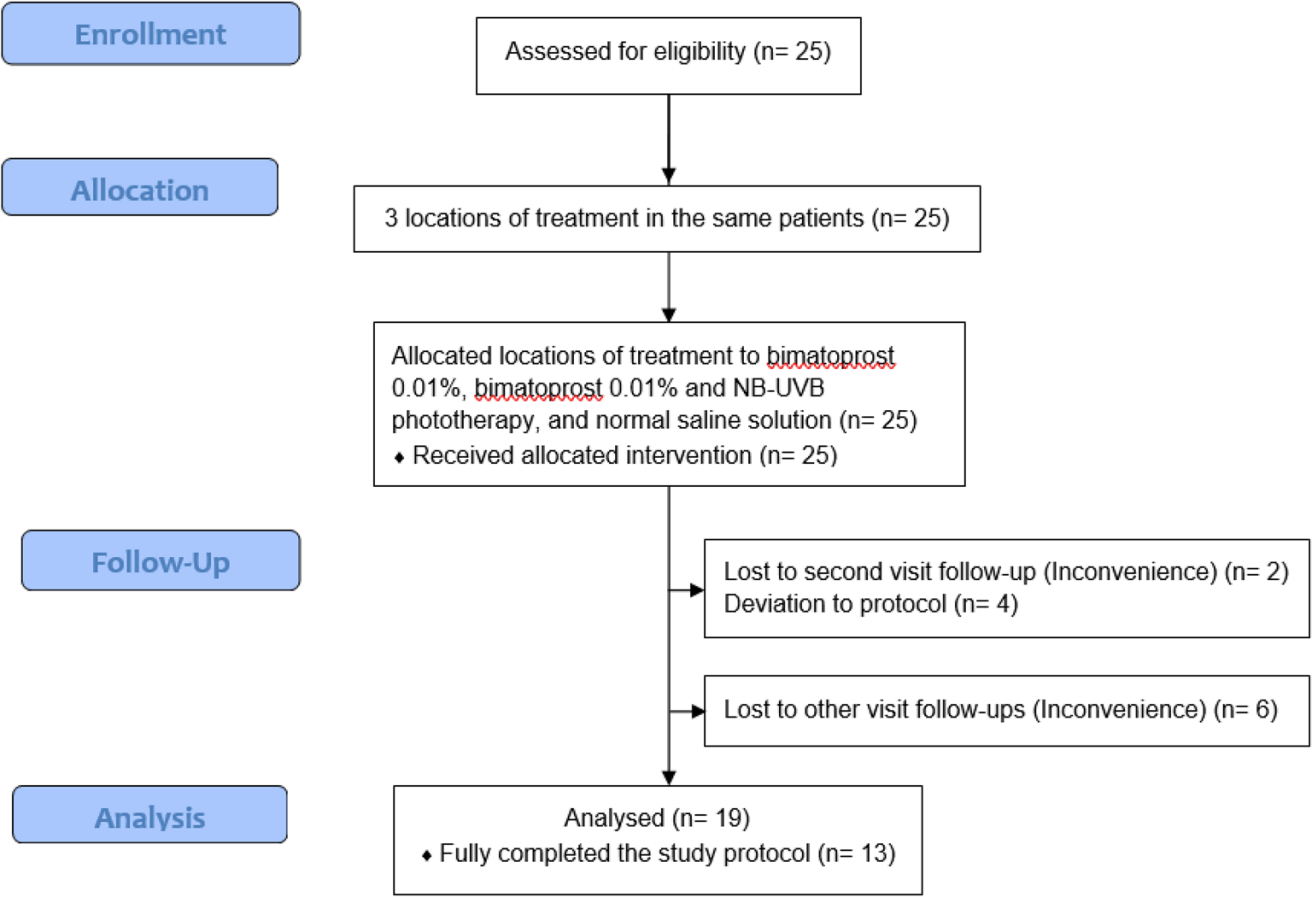
Diagram describing the flow of patients in this study.

A total of 25 subjects were initially enrolled in this study however, six subjects withdrew or were withdrawn from the study due to either inconvenience to receive phototherapy (n = 2) or deviation from the study protocol during the study (n = 4). The remaining 19 patients were analyzed. A diagram showing the flow of subjects in this study is shown in Fig. 1.

### Recruitment

All subjects were recruited in the study from March 2017 to April 2021 and the last follow up was in November 2021. The trial ended in November 2021 after 25 participants were recruited.

### Baseline data/numbers analyzed

Among the 19 analyzed subjects, the median (minimum, maximum) age was 35 years (14, 53), and the median duration of disease was 4 years (0.1, 23). Most participants were middle-aged females (63.2%). There were 13 cases of non-segmental vitiligo, and 6 cases of segmental vitiligo. Eight cases had face/neck lesions, and eleven cases had non-face/neck lesion. The baseline demographic and clinical characteristics of study patients are described in Table 1.

**Table 1:** Baseline demographic and clinical characteristics of vitiligo patients (N = 19).

Characteristics	Total (N = 19)	Non-segmental type (N = 13)	Segmental type (N = 6)
Gender, n (%)			
Male	7 (36.8%)	4 (30.8%)	3 (50.0%)
Female	12 (63.2%)	9 (69.2%)	3 (50.0%)
Age (years), median (min, max)	35 (14.0, 53.0)	35 (14.0, 53.0)	36 (24.0, 47.0)
Age of disease onset (years), median (min, max)	30 (7.0, 50.0)	30 (10.0, 50.0)	32 (7.0, 45.0)
Duration of disease (years), median (min, max)	4 (0.1, 23.0)	4 (0.1, 22.0)	2 (0.4, 23.0)
Site of intervention^a^, n (%) (n = 57)			
Face and neck	24 (42.1%)	15 (38.5%)	9 (50.0%)
Trunk	15 (26.3%)	12 (30.7%)	3 (16.7%)
Buttock	3 (5.3%)	3 (7.7%)	0
Extremity	9 (15.8%)	3 (7.7%)	6 (33.3%)
Axilla	6 (10.5%)	6 (15.4%)	0
Associated disease, n (%)			
Yes^b^	3 (15.8%)	3 (23.1%)	0
No	16 (84.2%)	10 (76.9%)	6 (100.0%)

^a^Each patient had three sites of intervention (1 placebo, 1 monotherapy, and 1 combination therapy).

^b^Among the three patients, two had hyperthyroid disease and one had hypothyroid disease.

### Outcomes and estimation

**Figure 2:**
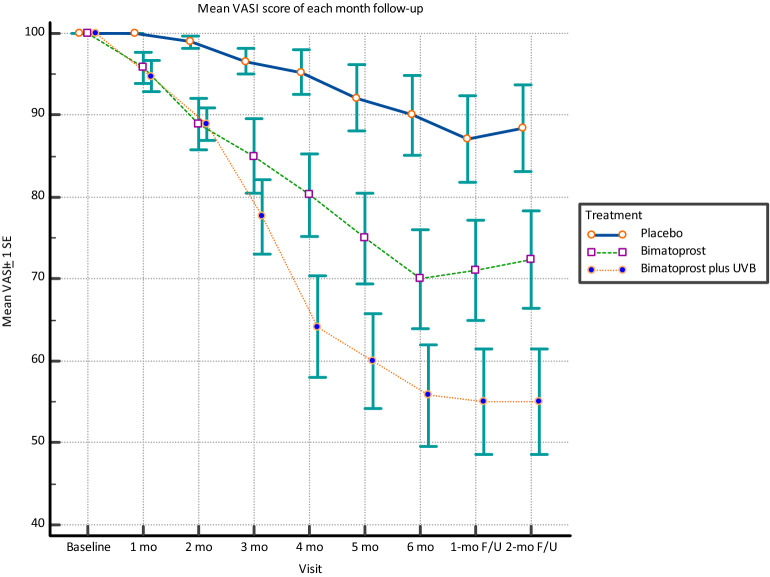
The mean Vitiligo Area Scoring Index (VASI) score with standard error bars (SE) at each monthly study visit, and at the 1-month and 2-month follow-up (F/U) visits compared among the placebo, bimatoprost, and bimatoprost plus narrowband ultraviolet B (NB-UVB) groups.

**Figure 3:**
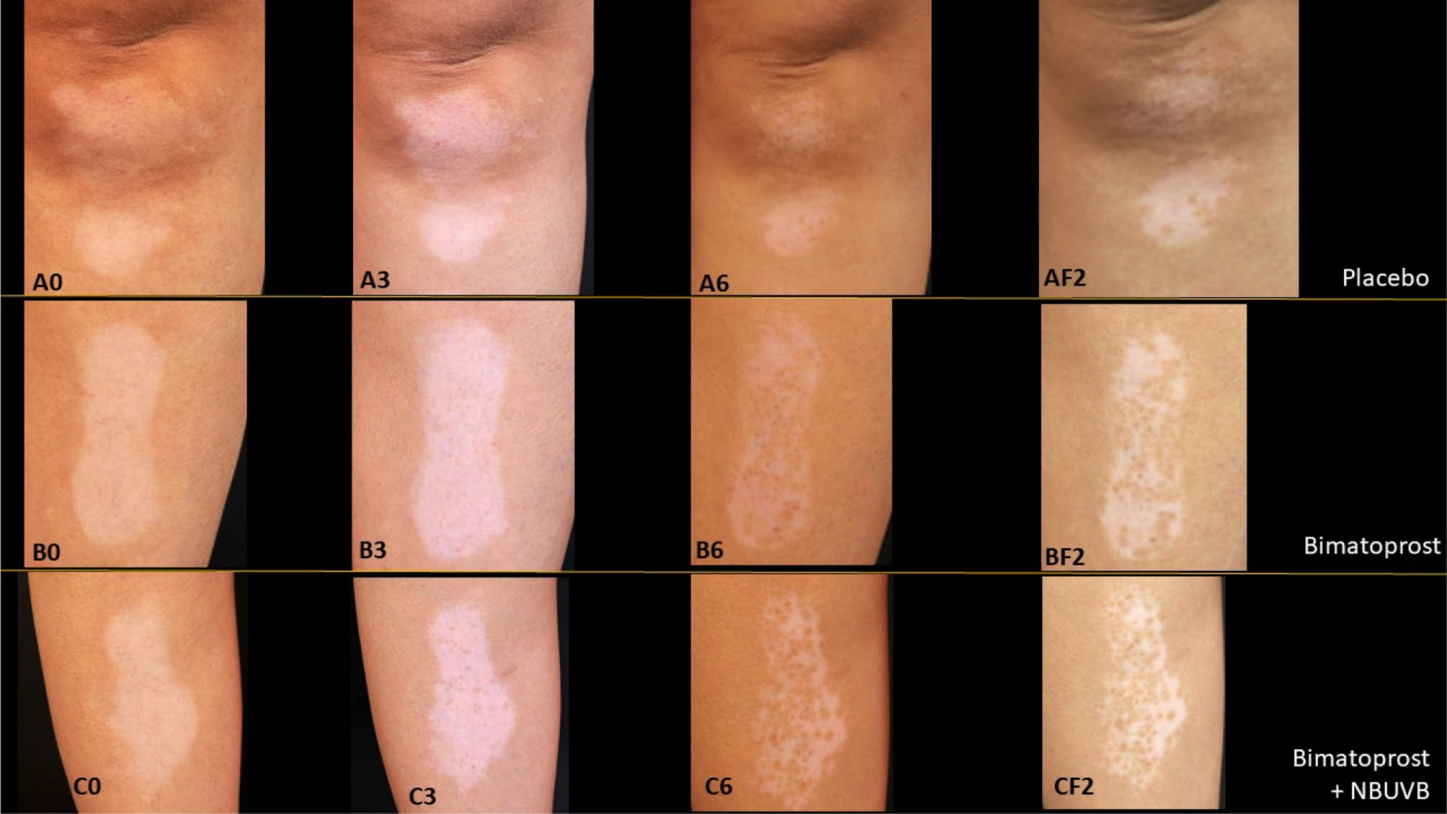
Repigmentation outcome in non-segmental vitiligo, both lower leg lesions. (**A**)  Placebo group: (**A0**) baseline, (**A3**) 3 months, (**A6**) 6 months, (**AF2**) 2 months follow-up. (**B**)  Bimatoprost monotherapy group: (**B0**) baseline, (**B3**) 3 months, (**B6**) 6 months, (**BF2**) 2 months follow-up. (**C**)  Combination therapy: (**C0**) baseline, (**C3**) 3 months, (**C6**) 6 months, (**CF2**) 2 months follow-up.

**Figure 4:**
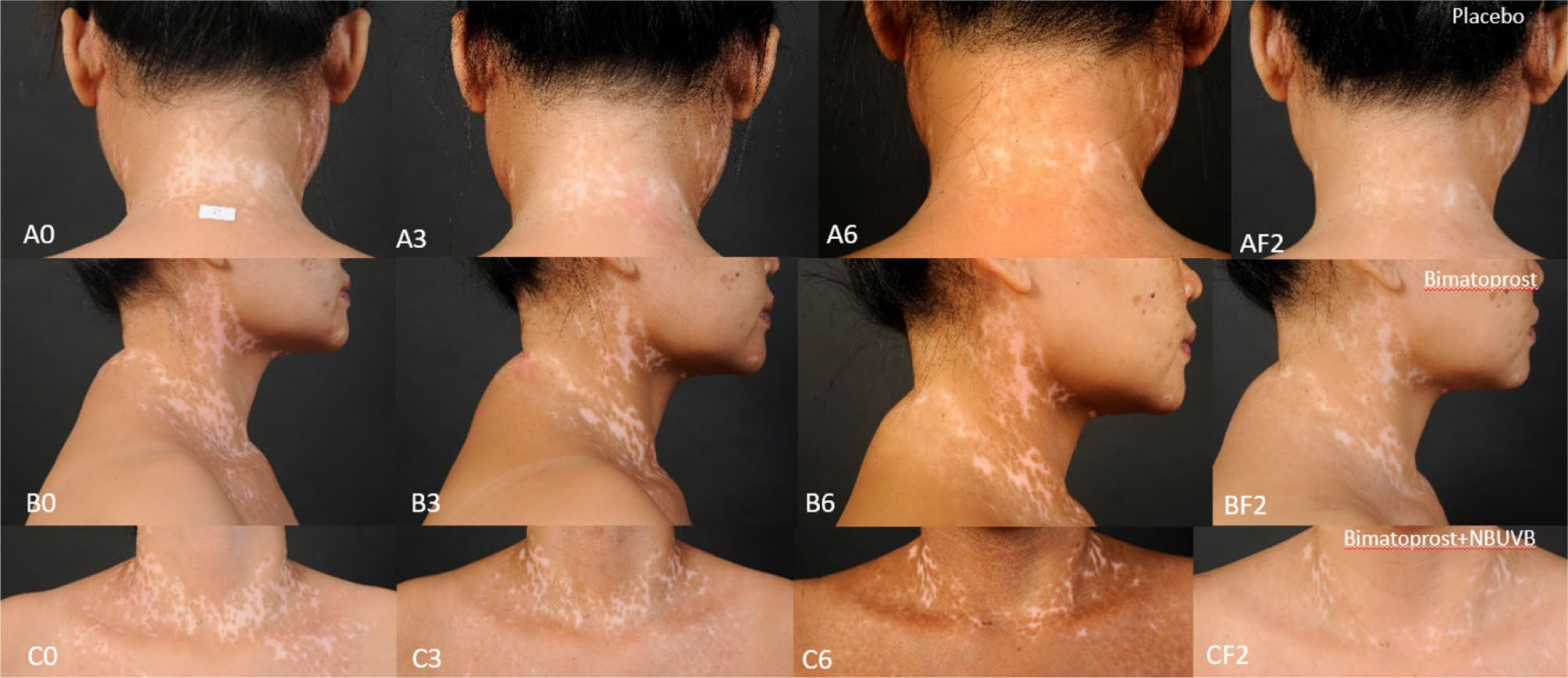
Repigmentation outcome in non-segmental vitiligo, neck lesions (**A**)  Placebo group: (**A0**) baseline, (**A3**) 3 months, (**A6**) 6 months, (**AF2**) 2 months follow-up. (**B**) Bimatoprost monotherapy group: (**B0**) baseline, (**B3**) 3 months, (**B6**) 6 months, (**BF2**) 2 months follow-up. (**C**)  Combination therapy: (**C0**) baseline, (**C3**) 3 months, (**C6**) 6 months, (**CF2**) 2 months follow-up.

Of 19 subjects, the mean ± standard deviation VASI score at each study and follow-up time point compared among the placebo, monotherapy, and combination therapy groups are shown in Table 2. The VASI score improved in both the monotherapy and combination therapy groups. Regarding statistical differences between study medications versus placebo at each study and follow-up time point, the VASI score was significantly different between the monotherapy group and the placebo group only at the 2-month time point. In contrast, the VASI score was significantly different between the combination therapy group and the placebo group at all study and follow-up time points. There was no statistically significant difference in VASI score improvement between monotherapy and combination groups. The mean VASI score at each monthly study visit, and the 1-month and 2-month follow-up (F/U) visits compared among the placebo, bimatoprost, and bimatoprost + NB-UVB groups are shown in Fig. 2. Although both bimatoprost interventions performed better than saline solution placebo, the bimatoprost combination intervention performed better than bimatoprost monotherapy. (Figs. 3, 4) Non-segmental type and non-face/neck lesions showed statistically significant improvement in VASI score in the combination therapy group when compared to placebo at the 3-month treatment time point, the 6-month treatment time point, and the 2-month follow-up time point after the cessation of treatment, as shown in Tables 3, 4, and 5, respectively. In addition, at 6 months, bimatoprost monotherapy group demonstrated significant improvement in VASI score in non-segmental type and non-face/neck lesions. (Table 4) No adverse effects from topical bimatoprost 0.01%, such as drug sensitivity, photosensitivity, or localized hypertrichosis, were observed or reported in this study.

**Table 2:** Mean ± standard deviation VASI score at each study and follow-up time point compared among the placebo, monotherapy, and combination therapy groups (ITT analysis).

Time point	n	VASI score			p*
		Placebo	Bimatoprost 0.01% monotherapy	Bimatoprost 0.01% + NB-UVB	
Baseline	19	100.00 ± 0.0	100.00 ± 0.0	100.00 ± 0.0	N/A
1 month	19	100.00 ± 0.0	95.79 ± 8.2	94.74 ± 8.2	0.028^b^
2 months	19	98.95 ± 3.2	88.95 ± 13.5	88.95 ± 8.4	0.001^a,b^
3 months	19	96.58 ± 6.7	85.00 ± 19.7	77.63 ± 19.9	0.003^b^
4 months	19	95.26 ± 11.7	80.26 ± 22.0	64.21 ± 26.8	0.001^b^
5 months	19	92.11 ± 17.6	75.00 ± 24.2	60.00 ± 25.4	0.001^b^
6 months	19	90.00 ± 21.0	70.00 ± 26.4	55.79 ± 26.8	0.001^b^
Follow up at 1 month	19	87.11 ± 23.1	71.05 ± 26.6	55.00 ± 27.9	0.002^b^
Follow up at 2 months	19	88.42 ± 23.1	72.37 ± 26.2	55.00 ± 27.9	0.002^b^

*VASI score* Vitiligo Area Scoring Index score, *NB-UVB* narrowband ultraviolet B phototherapy, *ITT* intention to treat.

*Repeated measurement ANOVA with multiple comparison by Bonferroni method; statistical significance defined as *p*-value < 0.05.

^a^Placebo vs monotherapy.

^b^Placebo vs combination therapy.

**Table 3:** Mean ± standard deviation VASI score for vitiligo type and lesion location at 3 months after the start of treatment compared among the placebo, monotherapy, and combination therapy groups (ITT analysis).

Type and location	n	Placebo	Bimatoprost 0.01% monotherapy	Bimatoprost 0.01% + NBUVB	p*
Type of vitiligo					
Non-segmental type	13	97.69 ± 4.4	83.46 ± 22.8	76.92 ± 17.3	***0.013***^b^
Segmental type	6	94.17 ± 10.2	88.33 ± 11.3	79.17 ± 26.5	0.357
Location of lesion					
Face and neck	8	91.88 ± 8.4	79.38 ± 23.5	79.37 ± 14.0	0.308
Other areas	11	100.00 ± 0.0	89.09 ± 16.3	76.36 ± 23.9	***0.008***^b^

A *p*-value < 0.05 indicates statistical significance.

*VASI score* Vitiligo Area Scoring Index score, *NBUVB* narrowband ultraviolet B phototherapy, *ITT* intention to treat.

*Repeated measures analysis of variance (ANOVA) with multiple comparisons by Bonferroni method.

^a^Placebo vs. monotherapy.

^b^Placebo vs. combination therapy.

**Table 4:** Mean ± standard deviation VASI score for vitiligo type and lesion location at 6 months after the start of treatment compared among the placebo, monotherapy and combination therapy groups (ITT analysis).

Type and location	n	Placebo	Bimatoprost 0.01% monotherapy	Bimatoprost 0.01% + NBUVB	p*
Type of vitiligo					
Non-segmental type	13	93.09 ± 9.3	70.77 ± 23.6	53.85 ± 27.8	** < *0.001***^a,b^
Segmental type	6	83.33 ± 36.1	68.33 ± 34.2	60.00 ± 26.5	0.490
Location of lesion					
Face and neck	8	80.63 ± 29.6	69.38 ± 25.6	53.13 ± 20.9	0.178
Other areas	11	96.82 ± 7.8	70.45 ± 28.2	57.73 ± 31.3	** < *0.001***^***a,***^^b^

A *p*-value < 0.05 indicates statistical significance.

*VASI score* Vitiligo Area Scoring Index score, *NBUVB* narrowband ultraviolet B phototherapy, *ITT* intention to treat.

*Repeated measures analysis of variance (ANOVA) with multiple comparisons by Bonferroni method.

^a^Placebo vs. monotherapy.

^b^Placebo vs. combination therapy.

**Table 5:** Mean ± standard deviation VASI score for vitiligo type and lesion location at the 2-month follow-up after the discontinuation of treatment compared among the placebo, monotherapy, and combination therapy groups (ITT analysis).

Type and location	n	Placebo	Bimatoprost 0.01% monotherapy	Bimatoprost 0.01% + NBUVB	p*
Type of vitiligo					
Non-segmental type	13	90.77 ± 15.4	73.85 ± 23.3	52.69 ± 29.3	** < *0.001***^b^
Segmental type	6	83.33 ± 39.4	69.17 ± 33.8	60.00 ± 26.5	0.496
Location of lesion					
Face and neck	8	78.13 ± 32.7	72.50 ± 24.3	53.13 ± 20.9	0.230
Other areas	11	95.91 ± 8.0	72.27 ± 28.6	56.36 ± 33.1	** < *0.001***^b^

A *p*-value < 0.05 indicates statistical significance.

*VASI score* Vitiligo Area Scoring Index score, *NBUVB* narrowband ultraviolet B phototherapy, *ITT* intention to treat.

*Repeated measures analysis of variance (ANOVA) with multiple comparisons by Bonferroni method.

^a^Placebo vs. monotherapy.

^b^Placebo vs. combination therapy.

## Discussion

Bimatoprost is a synthetic prostamide (prostaglandin-ethanolamides) F2α analogues. Prostaglandins are groups of lipid compounds that are produced from cells involved in inflammation, tissue damage, or infectious process. Previous studies showed that both PGE2 and PGF2α induced hyperpigmentation via the mechanism of melanocytic activation^9^. In vivo study suggested that the effects of PGF2α on melanocyte dendricity and tyrosinase activity may be accentuated by UV irradiation-dependent receptor regulation^10^. Subsequent in vitro study in guinea pigs found that all three analogues of PGF2α (latanoprost, bimatoprost, and travoprost) induced skin hyperpigmentation since pigmentation markedly increased when these agents were combined with NB-UVB^11^.

This randomized single-blind controlled clinical trial evaluated the efficacy of 0.01% topical bimatoprost ophthalmic solutions plus NB-UVB or bimatoprost 0.01% monotherapy compared to placebo in stable non-segmental or segmental vitiligo patients over an 8-month period. The VASI score was significantly improved in the combination therapy group compared to the placebo group at all 8 monthly time points, and repigmentation onset was observed as early as the 1-month. Although both the monotherapy and the combination therapy groups yielded better VASI score outcomes than placebo, the combination group outperformed the monotherapy group. There was, however, no statistically significant difference between the improvement in the VASI score compared between monotherapy and combination therapy at any time point in the study.

Previous studies of both latanoprost^8^ and bimatoprost^12–14^ in vitiligo showed effective repigmentation compared to placebo, even when using different concentrations of both agents. Anbar, et al. conducted a comparative study in non-segmental vitiligo and found a significant difference between latanoprost 0.005% monotherapy and placebo; however, latanoprost 0.005% monotherapy showed no significant difference compared to NB-UVB monotherapy. In addition, the combination of latanoprost 0.005% and NB-UVB yielded significantly better improvement in repigmentation compared to NB-UVB monotherapy^8^. A prospective study by Sharma, et al. found a statistically significant difference in mean percentage decreases in body surface area of vitiligo compared between bimatoprost 0.03% plus NB-UVB and NB-UVB monotherapy at 4, 8–16, and 24 weeks^15^. Furthermore, more than 50% repigmentation was achieved in 10 (40%) patients receiving NB-UVB alone compared to 13 (52%) patients in the combination treatment group (*p* > 0.05)^15^.

Comparing bimatoprost with other treatment, Kanokrungsee, et al., found a similar repigmentation result between bimatoprost 0.01% and tacrolimus 0.1% ointment in non-segmented facial vitiligo^14^. Grimes, et al. conducted a randomized, double-blind, controlled study of bimatoprost 0.03% monotherapy, bimatoprost plus mometasone, and mometasone plus placebo in non-facial vitiligo for a 20-week treatment period. Both bimatoprost groups achieved greater response compared with the mometasone plus placebo group by week 12^16^. In contrast, a study by Nowroozpoor, et al. in focal and generalized vitiligo found a similar Vitiligo Disease Activity (VIDA) score after 12 weeks of latanoprost 0.005% treatment compared to placebo^17^.

Regarding type of vitiligo, the results of our study demonstrated non-segmental vitiligo and non-face/neck lesions to be significant predictors of good response to combination therapy. Similarly, Kapoor, et al. conducted a study in different types of vitiligo (vulgaris, focal, segmental, and lip) using PGE2 (0.25 mg g^−1^) gel twice daily for 6 months, and the results showed better response in non-segmental and focal vitiligo^18^.

Concerning the location of vitiligo, Kapur, et al. reported that facial and scalp lesions responded earliest to vitiligo treatment with a mean onset of repigmentation of 1 to 1.5 months^19^. Jha, et al. reported that patients with facial vitiligo that were treated with bimatoprost 0.03% solution achieved more than 50% repigmentation by the end of week 12^13^. In the present study, face/neck lesions did not significantly respond to either monotherapy or combination therapy compared to placebo, which can be explained by the fact that most face/neck lesions in our study were segmental vitiligo, which was shown to have a lower response to bimatoprost when compared to non-segmental vitiligo.

The median duration of disease was 4 years, which is lower than the median disease duration reported from other studies, and this may result in earlier response to treatment. In addition to the overall efficacy of bimatoprost therapy demonstrated in the present study, our finding of significant response to treatment in the non-segmental vitiligo group is consistent with previously reported findings^12–14^. Moreover and importantly, we also found a significant improvement in VASI score in the combination therapy group compared to placebo among patients in the non-face/neck lesion group. This may be explained by the fact that most patients with non-face/neck lesions tended to have non-segmental vitiligo, which had a significantly better response to combination therapy when compared to segmental vitiligo.

Regarding the safety of bimatoprost ophthalmic solution for treating vitiligo, Jha, et al., reported localized hypertrichosis after 6 weeks of treatment at the periorbital area^20^. Another reported side effect was burning sensation that gradually resolved without added treatment. Kanokrungsee, et al. reported itching sensation in 30% of patients, burning sensation in 10% of patients, and hypertrichosis in one patient. However and in contrast, no adverse effects of topical bimatoprost were observed or reported in our study. In addition and importantly, we used a lower concentration of bimatoprost ophthalmic solution. The studies that reported adverse effect of treatment mostly used a concentration of 0.03%.

### Limitations

This study has some mentionable limitations. First, our data were collected from a single center. Moreover, our center is a large, university-based, national tertiary referral center that is routinely referred complex cases that are thought not to be sufficiently treatable at other levels of care. As such, some aspect of this study may not be immediately generalizable to vitiligo found in other care settings. Second, although we enrolled a number of subjects that satisfied the minimum number established by our sample size calculation, six of those patients were excluded due to inconvenience to remain in the study or deviation from the study protocol. Since our study analyzed less than the calculated minimum number of subjects, our study has lacked the statistical power needed to identify all statistically significant differences and associations between and among the three study interventions.

## Conclusion

Although monotherapy and combination therapy both outperformed placebo, combination therapy consisting of bimatoprost 0.01% and NB-UVB outperformed monotherapy, and was shown to be safe and effective for treating Thai patients with non-segmental vitiligo at areas other than the face/neck.

## Data Availability

The datasets used and/or analyzed during the current study are available from the corresponding author upon reasonable request.
